# The marker choice: Unexpected resolving power of an unexplored CO1 region for layered DNA barcoding approaches

**DOI:** 10.1371/journal.pone.0174842

**Published:** 2017-04-13

**Authors:** Jessica Rach, Tjard Bergmann, Omid Paknia, Rob DeSalle, Bernd Schierwater, Heike Hadrys

**Affiliations:** 1 ITZ, Ecology & Evolution, TiHo Hannover, Hannover, D-30559, Germany; 2 Sackler Institute of Comparative Genomics, American Museum of Natural History, New York, NY 10024, United States of America; Sichuan University, CHINA

## Abstract

The potential of DNA barcoding approaches to identify single species and characterize species compositions strongly depends on the marker choice. The prominent “Folmer region”, a 648 basepair fragment at the 5’ end of the mitochondrial CO1 gene, has been traditionally applied as a universal DNA barcoding region for metazoans. In order to find a suitable marker for biomonitoring odonates (dragonflies and damselflies), we here explore a new region of the CO1 gene (CO1B) for DNA barcoding in 51 populations of 23 dragonfly and damselfly species. We compare the “Folmer region”, the mitochondrial ND1 gene (NADH dehydrogenase 1) and the new CO1 region with regard to (i) speed and reproducibility of sequence generation, (ii) levels of homoplasy and (iii) numbers of diagnostic characters for discriminating closely related sister taxa and populations. The performances of the gene regions regarding these criteria were quite different. Both, the amplification of CO1B and ND1 was highly reproducible and CO1B showed the highest potential for discriminating sister taxa at different taxonomic levels. In contrast, the amplification of the “Folmer region” using the universal primers was difficult and the third codon positions of this fragment have experienced nucleotide substitution saturation. Most important, exploring this new barcode region of the CO1 gene identified a higher discriminating power between closely related sister taxa. Together with the design of layered barcode approaches adapted to the specific taxonomic “environment”, this new marker will further enhance the discrimination power at the species level.

## Introduction

DNA barcodes, short DNA sequences of a standardized gene region, have been highly promoted for their fast and reliable identification of specimens of unknown species origin. Numerous groups all over the world have been compiling efforts to construct a comprehensive DNA barcode database covering a major part of the worlds biodiversity [1–4]. More recent metabarcoding techniques have emerged to explore and monitor species composition in different environments (e.g. [5–8]). All of these approaches have in common that their potential (accuracy) in delimiting species strongly depends on DNA marker selected. Finding and applying appropriate markers for a specific “environment” is still a challenge and subject of ongoing discussions [9, 10].

The mitochondrial CO1 gene region (cytochrome c oxidase 1) has become the standard genetic marker for a broad range of animal phyla since it has been promoted as the “universal” DNA barcoding marker for Metazoa [11]. The CO1 gene bears some characteristics making it particularly effective for evolutionary studies. First, the size and structure of this mitochondrial gene appear to be conserved in aerobic organisms [12]. Second, the approximately 1600 basepair (bp) gene comprises a range of different functional domains showing heterogeneous substitution patterns [12–14]. Mitochondrial genes in general have several strong advantages as molecular markers. They are easy to amplify due to the high copy numbers per cell and their haploid character. They also evolve much faster than the coding regions of nuclear genes because mitochondria lack a proofreading mechanism (e.g. [15–17]). As for the emerging metabarcoding approaches the scientific community recently has expressed mixed “feelings” about mitochondrial markers, but overall mt-gene fragments might still be the markers of choice. Especially the rapid degradation of environmental DNA due to UV light and microbial activity [18] make short sequence fragments more likely to persist long enough for pooled species detection. Here, the larger copy number of mitochondrial genes seems to outweigh the disadvantages.

The international DNA barcoding initiative initially agreed upon a 650 bp fragment at the 5’end of the CO1 gene—the “Folmer region”—because it is flanked by “universal” primers that have successfully been employed for various metazoan taxa [19]. The idea of using a standard marker for DNA barcoding that eases the coordination of multiple research groups and the construction of a comprehensive reference library is still very lively but also quite ambitious. The crucial parameter for the choice of a marker fragment is the substitution rate. The patterns of molecular evolution within the CO1 gene have not yet been sufficiently studied and evolutionary rates of mitochondrial genes are known to vary extremely within and between taxa [20]. Thus, it is not remarkable that the “Folmer region” of CO1 performed well for the identification and assignment of samples in some taxonomic groups (for example birds [21–23], fishes [24, http://www.fishbol.org] and mammals [25, http://www.mammaliabol.org]) but failed in various other groups (bilaterian animals [26, 27], gastropods and amphibians [28]), and a wide range of marine invertebrate (e.g. [29]) and insects (e.g. [30]). More interestingly, in most studies using this partition of the CO1 gene, taxon specific primers have been used instead of the universal primers established by Folmer et al. in 1994 [29–32]. Besides these “drawbacks”, in August 2015 the iBOL (international Barcode of Life) consortium completed gathering barcodes from five million specimens, representing 500,000 species (see http://www.ibol.org).

In odonates, the “Folmer region” for DNA barcoding has been tested before [33]. It was shown that overlaps of intra- and interspecific variation were prevalent complicating the identification through genetic distances. Moreover, amplification success was limited and potential pseudogenes were co-amplified with the universal primers. Consequently, the addition of the mitochondrial ND1 gene region (NADH dehydrogenase subunit 1) has been used for character-based DNA barcoding in odonates to overcome these limitations [33–37].

It has become obvious that a layered barcode approach, i.e. adding a second, a third or even more additional markers to enhance the discrimination potential in many, and particularly metabarcoding studies, is highly desirable. In search for a more suitable marker to monitor biodiversity patterns in odonates (which are prominent freshwater-bioindicators) we explore a new marker and test it against the traditional ones. We evaluate a new partition of the CO1 gene (CO1B), an approximately 650 bp fragment downstream of the “Folmer region”, for its potential to reliably discriminate 23 odonate species of twelve genera and six families. Yet another fragment downstream of the “Folmer region” has successfully been used for discriminating species of three New Zealand damselfly genera [38]. It was also shown that this part of the gene was promising for DNA barcoding in sponges and presumably other diploblasts while the 5’region failed in these animal groups due to extremely low genetic divergences [13]. We here compare the CO1B partition with the “Folmer region” and the ND1 marker with regard to (i) the straightforwardness of amplification, sequencing and alignment procedures, (ii) the base composition and homoplasy level and (iii) the suitability for character-based DNA barcoding and its overall discriminating power for potential layered or metabarcoding approaches.

## Materials and methods

### DNA extraction, PCR and sequencing

Tissue samples of 130 individuals representing 23 species, 12 genera and 6 families were collected from 2001 to 2006 mostly by non-invasive sampling [39] and stored in 70% or 98% ethanol until DNA extraction. A summary of all analyzed species is given in Tables 1 and 2. Samples from South Africa were collected by Sandra Damm and Frank Suhling within the project BMBF Biota South S08. Samples from East Africa were collected by Viola Clausnitzer within the project BMBF Biota East E09.

**Table 1:** Information on anisoptera odonate samples.

Family	Species	Locality	Country	ID/Sequences	No. Ind.	GPS	Authority
Aeshnidae	*Aeshna cyanea*	Hannover	Germany	Acy1—Acy2, Acy4	3	52°21’ N / 09°48’ E	1
		Meißendorf	Germany	Acy03A	1	52°72’ N / 09°82’ E	1
Aeshnidae	*Aeshna grandis*	Hannover	Germany	Aegr05A	1	52°21’ N / 09°48’ E	1
Aeshnidae	*Aeshna mixta*	Hannover	Germany	Ami2—Ami3	2	52°21’ N / 09°48’ E	1
Aeshnidae	*Aeshna rileyi*	Kilimanjaro, Machame, Semira Riv.	Tanzania	Aeri142	2	03°10’ S / 37°13’ E	2
Aeshnidae	*Anaciaeschna triangulifera*	Pangani River	Tanzania	Anatri162	1	04°37’ S / 38°00’ E	2
Aeshnidae	*Anax imperator*	Tsaobis	Namibia	Ai21	3	22°31’ S / 15°50’ E	3
		Tsauchab River	Namibia	Ai16	4	24°30’ S / 16°06’ E	3
		Erb	Namibia	Ai61	1	22°38’ S / 14°38’ E	3
		Baynes Mts.	Namibia	Ai98	3	17°01’ S / 12°39’ E	3
Aeshnidae	*Anax speratus*	Naukluft	Namibia	As11	4	24°15’ S / 16°14’ E	3
		Tsauchab River	Namibia	As16	2	24°30’ S / 16°06’ E	3
Aeshnidae	*Brachytron pratense*	Braunschweig	Germany	Brpr02	2	52°15’ N / 10°30’ E	1
Aeshnidae	*Gynacantha villosa*	Arabuke Sokoke Forest	Kenya	Gyvill60	1	03°18’ S / 39°59’ E	2
Gomphidae	*Paragomphus genei*	Palmwag	Namibia	Pg3	3	17°22’ S / 12°15’ E	3
		Baynes Mts.	Namibia	Pg98	2	17°01’ S / 12°39’ E	3
Libellulidae	*Crocothemis erythraea*	Palmwag	Namibia	Ce3	3	19°53’ S / 13°56’ E	3
		Tsauchab River	Namibia	Ce7	1	24°30’ S / 16°06’ E	3
		Ongongo	Namibia	Ce32	3	19°08’ S / 13°49’ E	3
Libellulidae	*Crocothemis sanguinolenta*	Ongongo	Namibia	Cs7	3	19°08’ S / 13°49’ E	3
		Baynes Mts.	Namibia	Cs98	3	17°01’ S / 12°39’ E	3
Libellulidae	*Orthetrum julia falsum*	Tsauchab River	Namibia	Oj16	5	24°30’ S / 16°06’ E	3
		Waterberg	Namibia	Oj32	5	20°25’ S / 17°15’ E	3
Libellulidae	*Orthetrum trinacria*	Van-Bach-Dam	Namibia	Ot1	2	22°00’ S / 16°57’ E	3
		Palmwag	Namibia	Ot3	3	19°53’ S / 13°56’ E	3
Libellulidae	*Trithemis morrisoni*	Popa Falls	Namibia	Tst119	5	18°70’ S / 21°34’ E	3
Libellulidae	*Trithemis palustris*	Kwando	Namibia	Tst128	4	18°00’ S / 23°18’ E	3
					72		

The species and respective family names are given. The sample sites (Locality) and countries as well as the number of analysed individuals per locality are listed for each species. Authority: 1 = no specific permissions were required, no endangered or protected species were collected; 2 = BMBF Biota East E09; 3 = BMBF Biota South S08.

**Table 2:** Information on zygoptera odonate samples.

Family	Species	Locality	Country	ID/Sequences	No. Ind.	GPS	Authority
Coenagrionidae	*Pseudagrion acaciae*	Pangani River	Tanzania	Pa81	3	04°37’ S / 38°00’ E	2
Coenagrionidae	*Pseudagrion bicoerulans*	Mt.Elgon, Rongai River	Kenya	Pb77	4	01°02’ S / 34°46’ E	2
		Aberdare Mts, River	Kenya	Pb78	4	00°31’ S / 36°43’ E	2
		Kilim.,Machame,Semira Val.	Tanzania	Pb79	3	03°10’ S / 37°13’ E	2
		Mt.Kenya, Loruku	Kenya	Pb113	4	00°09’ S / 37°07’ E	2
Coenagrionidae	*Pseudagrion kersteni*	Naukluft	Namibia	Pk11	1	24°15’ S / 16°14’ E	3
		Kiboko River, Hunter‘s	Kenya	Pk72	2	02°15’ S / 37°21’ E	2
		Tsavo West, Mzima	Kenya	Pk73	2	02°58’ S / 38°01’ E	2
		Rufiji, Kichi Stream	Tanzania	Pk88	1	08°15’ S / 38°37’ E	2
		Usamb.Mts,Amani Pond	Tanzania	Pk94	3	05°05’ S / 38°37’ E	2
		Baynes Mts	Namibia	Pk98	2	17°01’ S / 12°39’ E	3
Coenagrionidae	*Pseudagrion massaicum*	Van-Bach-Dam	Namibia	Pm1	1	22°00’ S / 16°57’ E	3
		Kuiseb River	Namibia	Pm15	2	24°30’ S / 16°06’ E	3
		Tsauchab River	Namibia	Pm16	2	22°00’ S / 16°37’ E	3
		Shimba Hills, Pemba	Kenya	Pm37	5	04°11’ S / 39°24’ E	2
		Kiboko River, Hunter‘s	Kenya	Pm72	1	02°15’ S / 37°21’ E	2
Coenagrionidae	*Pseudagrion niloticum*	Tsavo West, Mzima	Kenya	Pn73	1	02°58’ S / 38°01’ E	2
		Kiboko River	Kenya	Pn72	1	02°15’ S / 37°21’ E	2
		Ewaso, NyiroRiv., Nguruman	Kenya	Pn76	4	01°78’ S / 36°13’ E	2
Protoneurinae	*Chlorocnemis abbotti*	Uluguru Mts,Pandanus For.	Tanzania	Ca54	1	07°01’ S / 37°48’ E	2
		Udzungwa Mts, Sonje	Tanzania	Ca55	1	07°45’ S / 36°53’ E	2
		Kilim.,Machame,Semira Val.	Tanzania	Ca79	5	03°10’ S / 37°13’ E	2
		Uzamb.Mts,Amani,Sigi Val.	Tanzania	Ca83	1	05°05’ S / 38°39’ E	2
Pseudostigmatidae	*Coryphagrion grandis*	Arabuke Sokoke Forest	Kenya	Cg19	2	03°18’ S / 39°59’ E	2
		Bandas, Shimba Hills	Kenya	Cg22	2	04°12’ S / 39°27’ E	2
					58		

The species and respective family names are given. The sample sites (Locality) and countries as well as the number of analysed individuals per locality are listed for each species. Authority: 2 = BMBF Biota East E09; 3 = BMBF Biota South S08.

Prior to the phenol chloroform DNA extraction after Hadrys et al. [39], the tissue was freeze-dried with liquid nitrogen for a better homogenization. Sequences of ND1 and the “Folmer region” were obtained as described in Bergmann, Rach [33]. For the amplification of the CO1B fragment the newly designed primers OdoCO1Fw (5’>TACACGAGCATATTTTACTTCAGC>3’) and OdoCO1Rev (5’ >CTTAAATCCATTGCACTTTTC>3’) were used. The 25 *μ*l PCR reaction mixes contained 2.5 *μ*l of 10 X Taq DNA polymerase buffer (Bioline/Invitrogen), 2.5 mM MgCl_2_, 0.1 mM dNTPs, 7.5 pM each primer and 0.5 U Taq DNA polymerase (either Invitrogen or Bioline). Thermocycler conditions were initial denaturing at 95°C 3 min, 35 cycles of 30 s denaturing at 95°C, 30 s annealing at 53°C, 1 min extension at 72°C, followed by a final extension of 6 min at 72°C. PCR products were bidirectionally sequenced on a MegaBACE 500 sequencer using the DYEnamic ET Dye Terminator Cycle Sequencing Kit (Amersham Bioscience). Sequences were assembled and edited using SEQMANII (v. 5.03; DNASTAR, Inc.). All sequences were deposited in Genbank (CO1A&B: KY847543—KY847672; ND1: KY847673—KY847802).

### Alignment and sequence analyses

Consensus sequences of all samples and of the three fragments were aligned using MUSCLE [40]. The alignments were shortened to unambiguously alignable core regions of 541 bp (Folmer region), 508 bp (CO1B) and 335 bp (ND1). The alignment procedure was straightforward for the “Folmer region” and CO1B and no insertions or deletions (indels) were observed. The alignment of the ND1 sequences was more complex due to several indels at the 5’ -end of the sequences where parts of the 16S rDNA gene and the tRNA^Leu^ are located. All but one gap were removed by shortening the alignment to 335 bp. The only remaining gap is located at position 20. The gap was kept because one species (Pg- *Paragomphus genei*) has a characteristic “A” at this position. This insert has been observed for all five samples of this species and was unique to this group. Nucleotide base compositions and numbers of parsimony informative sites were quantified for all sites of each marker and for the three codon positions separately using DAMBE [41].

Phylogenetic trees were generated for each dataset by using parsimony in PAUP version 4.0b10 [42]. All characters were weighted equally and tree statistics were calculated with uninformative characters excluded. Heuristic searches using parsimony were performed with 100 random sequence-addition repetitions and TBR branch swapping. Consensus trees for the three datasets were computed to determine tree lengths and the homoplasy indices (HI).

To obtain a visual display of substitution saturation the number of transitions and transversions versus divergence was plotted for (i) all sites (ii) first and second codon position and (iii) only third codon positions using DAMBE [41]. The K2P substitution model was used as a measure of divergence because it accommodates transition/transversion rate bias. To construct a reproducible criterion for “saturation” a second-order polynomial regression line was fitted to the transition and transversion data of each saturation plot.

### CAOS analyses

CAOS (Character Attribute Organization System) was used to identify diagnostic characters for taxonomic groups. Here, diagnostic characters are pure characteristic attributes (CA) occurring only in one clade of a particular node [33, 37, 43, 44] within the strict consensus trees of the parsimony analyses. For comparison, examples of sister taxa pairs (genera, species or populations) were chosen and the numbers of pure CAs identified for each taxon were listed for all three markers (Table 3). The CAOS analyses were performed as described in Rach, Desalle [37] and Paknia, Bergmann [45].

**Table 3:** Diagnostic characters for sister taxa.

Taxon level	Taxon name		Diagnostic characters		
			CO1A	CO1B	ND1
Genus	*Aeshna*	*Anax*	6	19	5
Genus	*Crocothemis*	*Orthetrum*	21	18	17
Species	*Pseudagrion kersteni*	*P. bicoerulans*	43	49	22
Species	*Anax imperator*	*A. speratus*	29	41	6
Species	*Aeshna cyanea*	*A. mixta*	35	50	20
Population	*Orthetrum julia falsum* (Tsau.)	*O. julia falsum* (Wat.)	5	1	1
Population	*Pseudagrion bicoerulans* (Mt. Kenya)	*P. bicoerulans* (Mt. Elgon)	2	2	4
Population	*Pseudagrion kersteni* (Baynes Mts.)	*P. kersteni* (5 pop.)	0	13	4

Numbers of diagnostic characters for sister taxa of different taxonomic levels (genera, species and populations) identified within the three analysed genetic markers (“Folmer region”, CO1B and ND1).

## Results

### Sequence analyses

Amplification of the CO1B and ND1 fragments were successful for all species. The “Folmer region” could not be amplified for all three individuals of one species, *Paragomphus genei*, even after several retries (see also [33]). Consequently, this taxon was excluded from the study.

A compositional bias towards AT was observed for all three markers. The ND1 data set revealed the highest AT content followed by CO1B (ND1: 73.3%; CO1B: 68.3%, Folmer: 65.4%; see Table 4). The sequences of the “Folmer region” and ND1 showed the highest AT occurrence at the first codon position (Folmer: 86.5% (1st), 51% (2nd), 58.5% (3rd); ND1: 84.6% (1st), 64.9% (2nd), 69.8% (3rd)) while the highest AT content within the CO1B fragment was observed at the second and third codon positions (61.5% (1st), 72% (2nd), 71.4% (3rd)). All base compositions are summarized in Table 4.

**Table 4:** Sequence information.

Marker	(Codon Pos.)	T(U)	C	A	G	A/T	Parsimony informative (PI) sites/Total sites	% PI sites
**Folmer**	**(all sites)**	33,8	17,6	31,6	17	65,4	213/541	39,4
**CO1B**	**(all sites)**	36	16,7	32,3	14,9	68,3	245/508	48,2
**ND1**	**(all sites)**	47,3	11,3	26	15,4	73,3	172/335	51,3
**Folmer**	**(1st pos.)**	36	8,7	50,5	4,8	86,5	39/180	21,7
**CO1B**	**(1st pos.)**	35,2	19,9	26,5	18,4	61,7	111/169	65,7
**ND1**	**(1st pos.)**	57,4	4,3	27,6	10,6	85	47/112	42.0
**Folmer**	**(2st pos.)**	21,4	17,9	29,6	31,1	51	6/180	3,3
**CO1B**	**(2st pos.)**	39,8	17,7	32,2	10,3	72	44/170	25,9
**ND1**	**(2st pos.)**	37,7	13,8	27,2	21,3	64,9	25/112	22,3
**Folmer**	**(3st pos.)**	44	26,2	14,5	15,3	58,5	168/181	92,8
**CO1B**	**(3st pos.)**	33	12,5	38,4	16,1	71,4	90/170	52,9
**ND1**	**(3st pos.)**	46,6	15,8	23,2	14,4	61	100/112	89,3

The proportion of each base (%) at all sites and at only 1st, 2nd and 3rd codon positions is shown for the three analysed mitochondrial gene partitions. The percentage of Parsimony informative (PI) sites is listed for all sites and for 1st, 2nd and 3rd codon positions separately. The value was determined by dividing the number of PI sites to the total numbers of basepairs (Total sites).

The highest percentage of parsimony informative (PI) sites was found within the ND1 alignment (51.3%) followed by CO1B (48.2%). The dataset of the “Folmer region” revealed the lowest number of PI sites (39.4%). The great majority of sites of ND1 and the “Folmer region” are parsimony informative at third codon positions (ND1: 89.3%; Folmer: 92.8%). The CO1B fragment revealed however, the most PI sites at the first codon positions (65.7% of all 1st codon positions) and only 52.9% of the 3rd codon positions were parsimony informative.

The Maximum Parsimony analyses identified significantly different numbers of equally most-parsimonious trees for the three markers (ND1: 164, Folmer: 1558, CO1B: >10000). The Homoplasy Index (HI) which describes the proportion of character change in a data set that is homoplastic for a phylogenetic tree [46] was highest in the “Folmer” dataset (0.632) followed by CO1B (0.614) and lowest in ND1 (0.532).

Nucleotide substitution saturation was studied by surveying the shape of the second-order polynomial regression line that was fitted to transition and transversion data of each saturation plot. If the slope of this regression line was zero or negative the data were considered to be saturated. When all codon positions were analyzed together, no substitution saturation was observed in the three data sets (S1a–S1c Fig). The slopes of the graphs for transitions and transversions in the “Folmer” and CO1B saturation plots increased continuously with rising K2P distances. The gradient of the graph describing transitions in the ND1 saturation plot showed only a minimal increase when the pairwise K2P distances reached a value of approximately 0.25. Combined analyses of the first and second codon positions revealed no substitution saturation in all three data sets either (S1d and S1f Fig). When only third codon positions were examined, no saturation of transitions and transversions were detected in the CO1B and ND1 data sets (S1h and S1i Fig). The “Folmer” data set showed a saturation of transversions at a K2P distance value of 0.7 and above, while the graph of transitions increased steadily with rising K2P distances (S1g Fig).

### Character based analyses (CAOS)

The numbers of pure characteristic attributes (CAs, [44]) obtained for sister taxa by analyzing the three data sets with the CAOS algorithm are given in Table 3. At the genus level two examples were chosen for comparison of the three markers. The two species of the family Aeshnidae, *Aeshna* and *Anax*, can be discriminated by 19 pure CAs within the CO1B fragment, while only six and five diagnostic characters were found within the “Folmer region” and ND1. At the genus level twenty-one CAs within the “Folmer region”, 18 within CO1B and 17 within ND1 distinguish the libellulid genera *Crocothemis* and *Orthetrum*.

As for closely related sister species two pairs of the Aeshnidae were chosen, *Anax imperator*/*Anax speratus* and *Aeshna cyanea*/*Aeshna mixta*. For both pairs the CAOS analyses revealed the highest numbers of CAs within the CO1B region (41/50), followed by the “Folmer region” (29/35) and fewest CAs within ND1 (6/20). The same result was found for the sister species of the Coenagrionidae, *Pseudagrion kersteni* and *Pseudagrion bicoerulans* (CO1B: 49, “Folmer”: 43, ND1: 22).

For comparison of the three markers at the population level, three examples were chosen. Here, interestingly in all three cases a different marker revealed the highest number of diagnostic characters for distinguishing populations (Table 3). For example, for the Namibian population “Baynes Mountains” of *Pseudagrion bicoerulans* 13 pure CAs were found within CO1B and four within ND1 for distinguishing these samples from individuals of five other populations from Namibia, Kenya and Tanzania. Here, no pure CA was found within the “Folmer region” for the same comparisons.

## Discussion

The Odonata are a prominent order at the base of flying insects (Pterygota), the most species rich and important animal group on earth but—notoriously undetected—on the brink of mass extinction. Many pterygote orders need immediate attention. Monitoring their biodiversity patterns over time and space by reliable and fast DNA(meta)barcoding studies would be highly desirable. Hereby the choice of markers adapted to the “specific taxonomic environment” is the most important “genetic predisposition” to achieve this task. While the first study on DNA barcoding presented by Hebert, Cywinska [11] focused on the “Folmer region” and the “Consortium for the Barcode of Life” (CBoL) has adopted this part of the CO1 gene as universal DNA barcoding marker for species identification in a broad range of taxa (e.g. [47–50]); it has also been shown that the “Folmer region” did not deliver reliable DNA barcodes in other animal groups (e.g. various insect orders), due to low divergence rates or overlapping inter- and intraspecific genetic distances [13, 51–53]. Thus, identifying, testing and employing other DNA markers for specific taxa and/or various (meta)barcoding techniques and questions have become a “conditio sine qua non”.

### PCR anomalies and odonate DNA barcoding

Amplification success of a suitable barcoding region depends on the presence of conserved flanking regions that can serve as universal priming sites. While the “Folmer region” based universal primers have been working for many animal groups [19], for our datasets containing 130 odonate specimens the amplification success with these primers was moderate. Alteration of PCR conditions were necessary for various samples, and for one species no amplification products were obtained at all. This observation highlights the fast and different mutation ratios within and between species. Further studies of the Folmer fragment and its flanking regions in odonates revealed a lack of highly conserved domains [33], which made the design of odonate specific primers for this specific gene region particularly difficult. In contrast, the odonate specific primers that have been used for the CO1B fragment show “universally” excellent performance. PCR products were obtained easily for all species and specimen.

### Comparison of homoplasy levels of different mitochondrial gene fragments

Strong compositional biases cause saturation of nucleotide substitution [17, 54] and result in homoplasy when placed into a tree context. Homoplasy can lead to low genetic distances between taxa with deep divergence and might result in incorrect taxonomic assignments when distance-based methods are used, especially in cases of incomplete taxon sampling [55]. Comparative analyses of the behavior of different markers, using homoplasy as a guide, will reveal characteristics of the markers for their potential utility. In order to investigate levels of homoplasy we analyzed (i) base composition bias, (ii) distribution of parsimony informative (PI) sites (iii), numbers of most-parsimonious trees (MPTs), including their Homoplasy Indices (HI) and (iv) base substitution saturation in the three markers.

All three genetic markers showed a compositional bias towards AT. The AT content was highest in ND1 (73.3%) and lowest in the “Folmer region” (65.4%). The AT content at the third codon positions where generally most nucleotide substitutions occur was highest in CO1B (71.4%) and lowest in the CO1 “Folmer region” (58.5%). The number of parsimony informative (PI) sites within a fragment reflects its variability. Analyses of the distribution of PI sites at the codon positions might additionally indicate homoplasy levels. If most PI sites are restricted to one codon position high levels of homoplasy are likely. The highest percentage of PI sites was observed within the ND1 region (51.3%), followed by CO1B (48.2%). The “Folmer region” revealed only 39.4% PI sites. But, the great majority of third codon positions of the “Folmer region” and ND1 are parsimony informative (Folmer: 92.8%; ND1: 89.3%). The PI sites within the CO1B are distributed more evenly and 52.9% of the third codon positions are parsimony informative. As DNA codons are degenerated and in most cases the same amino acid is encoded by codons showing differences in the second or third codons, CO1B having most PI sites in the first codon position (65,7%) indicates higher barcoding potential than the “Folmer region”. Only three amino acids (Arg, Leu, Ser) out of twenty have codons varying the first codon position. Therefore, in CO1B the high PI at this position indicates meaningful changes of amino acid chain structures between specimens.

Parsimony analyses generally return a large number of equally most-parsimonious trees (MPTs). The parsimony analyses of CO1B revealed a high number of more than 10000 MPTs. The number of MPTs for ND1 and the “Folmer region” was much lower (ND1: 164; Folmer: 1558). The Homoplasy Index (HI) also was lowest in ND1 but highest in the “Folmer region”.

To test for substitution saturation, the numbers of transitions and transversions of each marker were plotted against pairwise K2P distances. Optimally the transitional and transversional substitutions should linearly increase with K2P. However, with the increase of divergence time, multiple substitutions at the same site might occur and the linear correlation for the character transformations is lost. No substitution saturation was observed when all codon positions of each marker were analyzed and when first and second codon positions were examined. However, when only third codon positions were analyzed, the “Folmer region” shows experienced saturation, while transitions increase linearly with the K2P model. The slopes of the regression lines for transitions and transversions at third codon positions of the ND1 fragment did not show a linear correlation using the K2P model. The graphs for transitions and transversion at the third codon positions of CO1B show a linear pattern. These results are congruent with the observed percentages of PI sites at the third codon positions.

In general, these results suggest that mitochondrial genes and, moreover, different partitions within mitochondrial genes, may highly differ in their nucleotide substitution rates and patterns [56]. For example, in odonates the third codon position of the “Folmer region” has experienced saturation of nucleotide substitutions and thus a decrease of genetic distances with increasing divergence times is likely. In contrast, within the more downstream partition of the CO1 gene, the CO1B region, additional PI sites were found. PI sites in the CO1B region were more evenly distributed at the three codon positions than within the “Folmer region”. The data did not indicate substitution saturation at any of the codon positions. Thus, the genetic distances of the CO1B show a higher correlation with odonate divergence times and consequently a more accurate assignment of close sister taxa than the barcode standard, although both regions are parts of the same mitochondrial gene.

### Diagnostic characters for sister taxa

In order to discriminate closely related taxa through discrete diagnostic characters, the CAOS algorithm was used to identify pure characteristic attributes (CAs) for pairs of sister taxa. Pure CAs are diagnostic characters that are present in one group but absent in the alternate group of a node within a guide tree [37, 43–45]. Combinations of pure CAs can serve as reliable character-based DNA barcodes for species and also for genera and populations [33, 37].

The CO1B fragment showed the highest number of diagnostic characters in most sister taxa comparisons as well as at different taxonomic levels. For example, the CAOS analysis revealed 19 diagnostic characters within the CO1B fragment for distinguishing the aeshnid genera *Aeshna* and *Anax*, but only six and five within the “Folmer region” and ND1. For the discrimination of the sister species *Aeshna cyanea* and *Aeshna mixta* 50 pure CAs were identified within CO1B, 35 within the “Folmer region” and 20 within ND1. The CO1B fragment also showed good performance in discriminating geographical entities and discrete populations. For the Namibian “Baynes Mountains” population of *Pseudagrion kersteni*, the CO1B sequences revealed 13 diagnostic characters to differentiate these samples from individuals of five other populations of this species from Namibia, Kenya and Tanzania. Here, only four diagnostic characters were found within ND1 and no pure CA within the “Folmer region”.

The numbers of diagnostic characters for discriminating closely related taxa is directly related to the suitability of the particular marker to deliver reliable DNA barcodes. In that sense the character-based barcoding approach has the potential to “filter” the discrimination power of a given marker at different taxonomic levels! At different taxonomic levels a character-based approach filters the informative signal for this level. A distance-based approach reduces all character information equally to distance values between two samples without consideration of taxon specific signals. Given that the Folmer sequences comprised 541, the CO1B sequences 508 and the ND1 sequences only 335 basepairs, the CO1B showed clearly the highest resolution power per base pair of sequence.

## Conclusion

The main criteria for the suitability of a genetic marker for DNA barcoding are (i) the simple isolation under various laboratory conditions, (ii) low levels of homoplasy and (iii) high numbers of diagnostic characters for the differentiation of sister taxa. In this paper we compared three discrete mitochondrial DNA fragments with regard to their potential for DNA barcoding in odonates. We show that the “Folmer region” (the barcode standard) revealed a high percentage of parsimony informative sites at the third codon positions and that transversions at these positions experience substitution saturation in odonate species comparisons. This saturation might lead to reduced genetic differentiation at higher taxonomic levels and consequently to false positive assignments of unknown samples when using this marker in DNA barcoding. The CO1B fragment showed the highest number of diagnostic characters for discriminating close sister taxa on different taxonomic levels. We suggest that this gene region is able to deliver reliable DNA barcodes for developing a fast monitoring approach in odonates in general. In summary, there are clear differences in the performance of DNA fragments considering different criteria important for DNA barcoding. We further suggest that a layered barcode including several markers will most likely increase the identification success and reliability of DNA barcodes in general.

## Supporting information

S1 Fig Second-order polynomial regression. Transition and transversion data of each saturation plot.
(PDF)
