# Supplementary figures and images for: The marker choice: Unexpected resolving power of an unexplored CO1 region for layered DNA barcoding approaches
Source: PLoS One. 2017 Apr 13;12(4):e0174842. doi: 10.1371/journal.pone.0174842 (PMC5390999; doi:10.1371/journal.pone.0174842)

## "Folmer region"

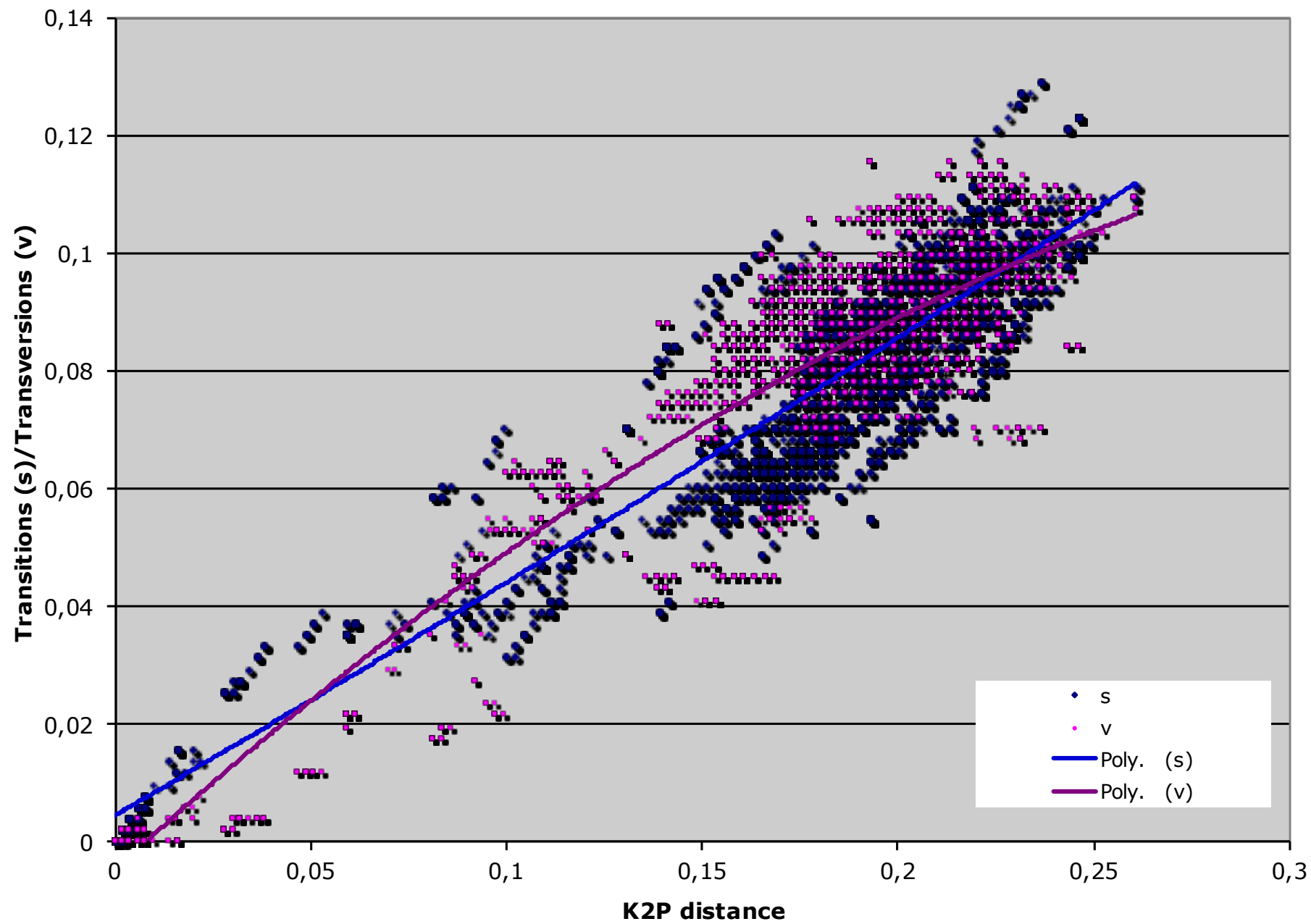

# CO1B

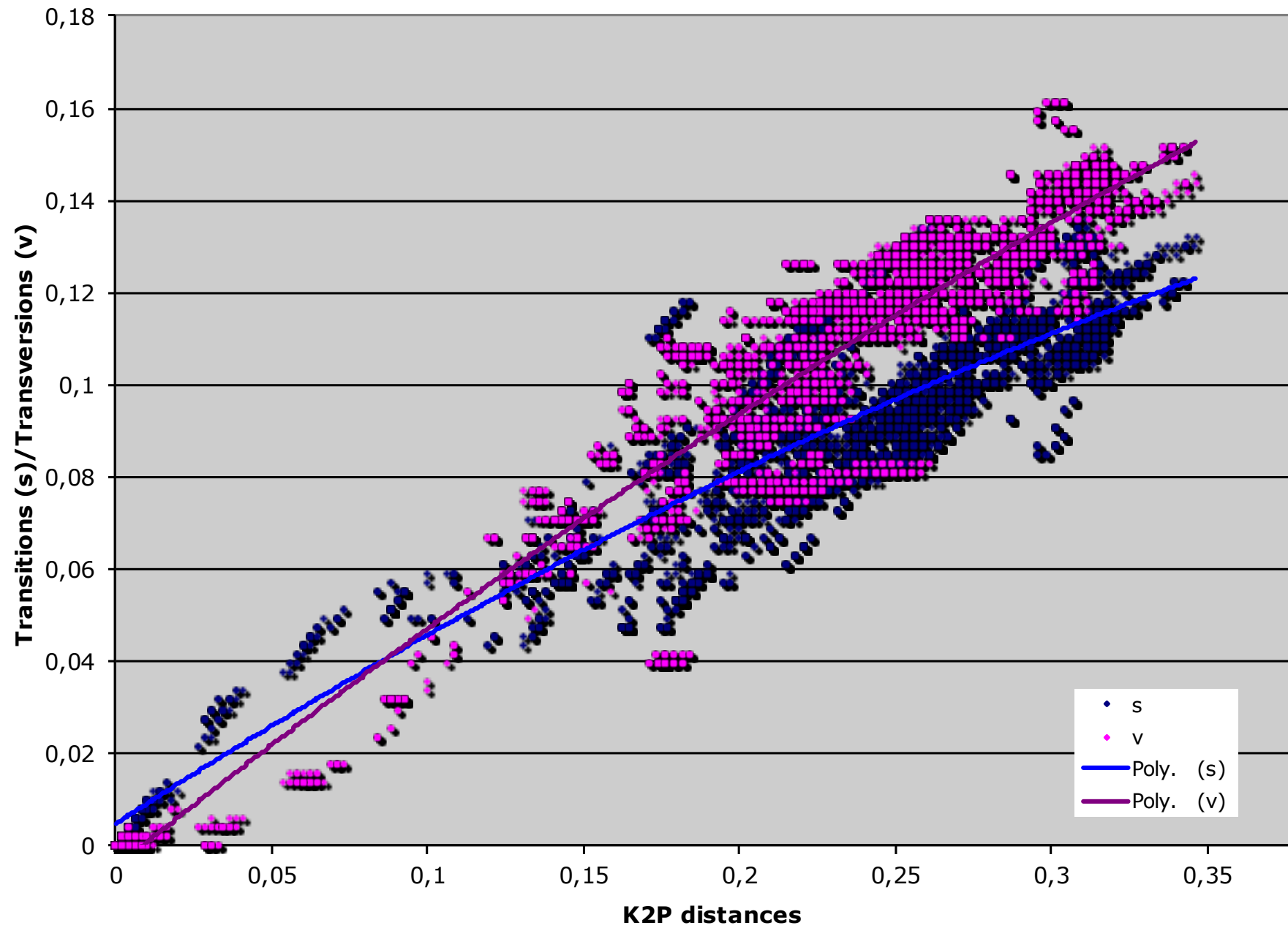

ND1

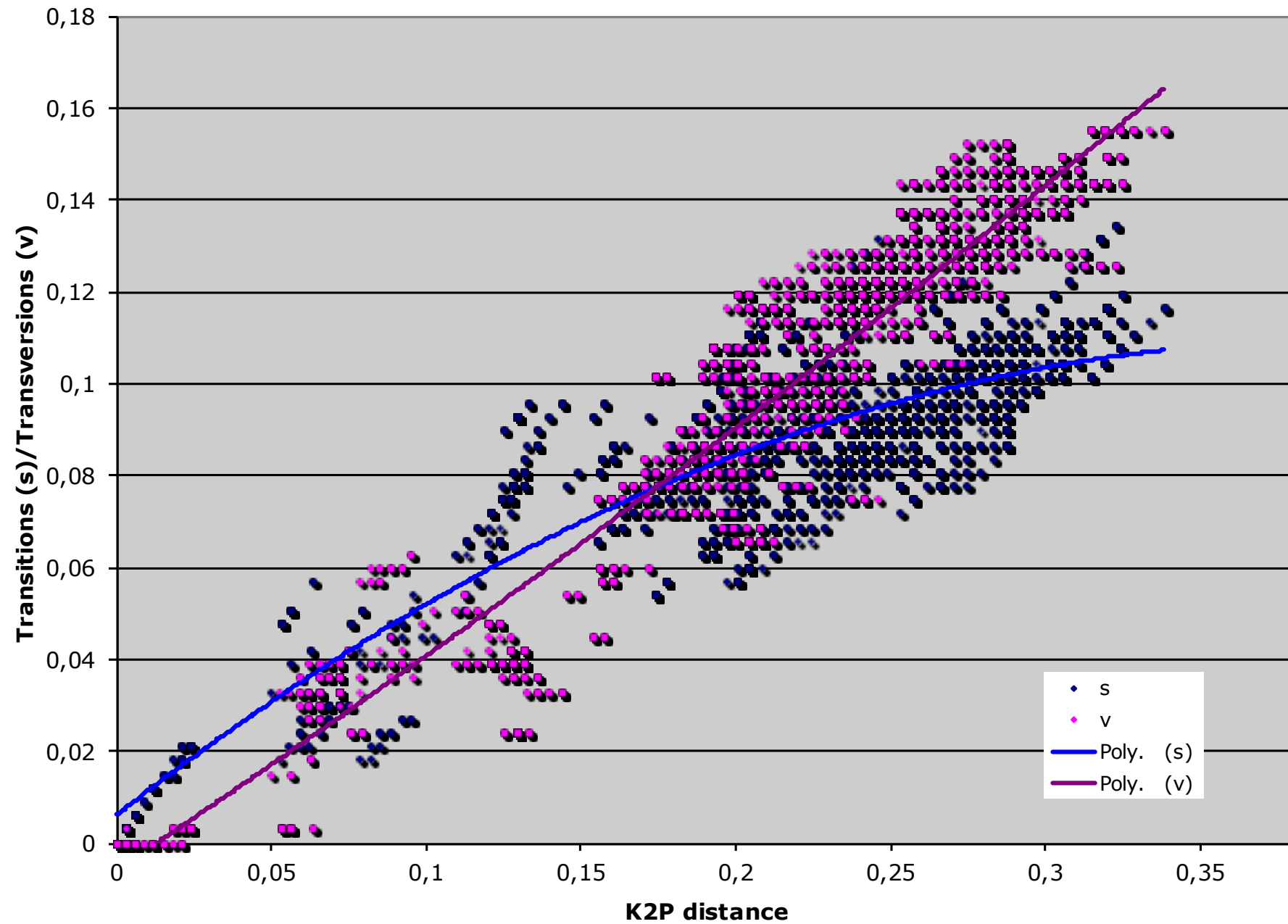

# Folmer 1st + 2nd Pos.

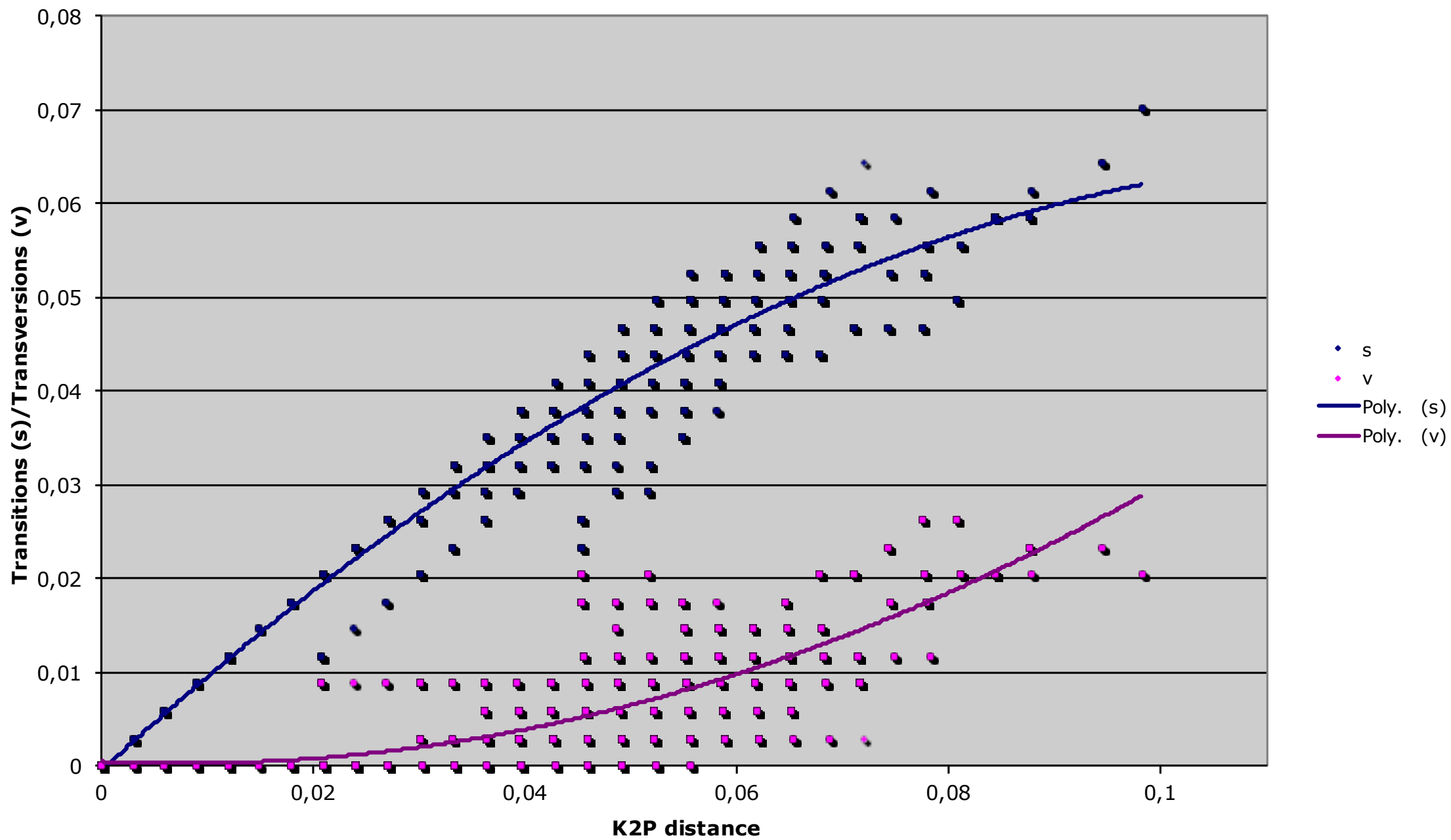

# CO1B 1st + 2nd Pos.

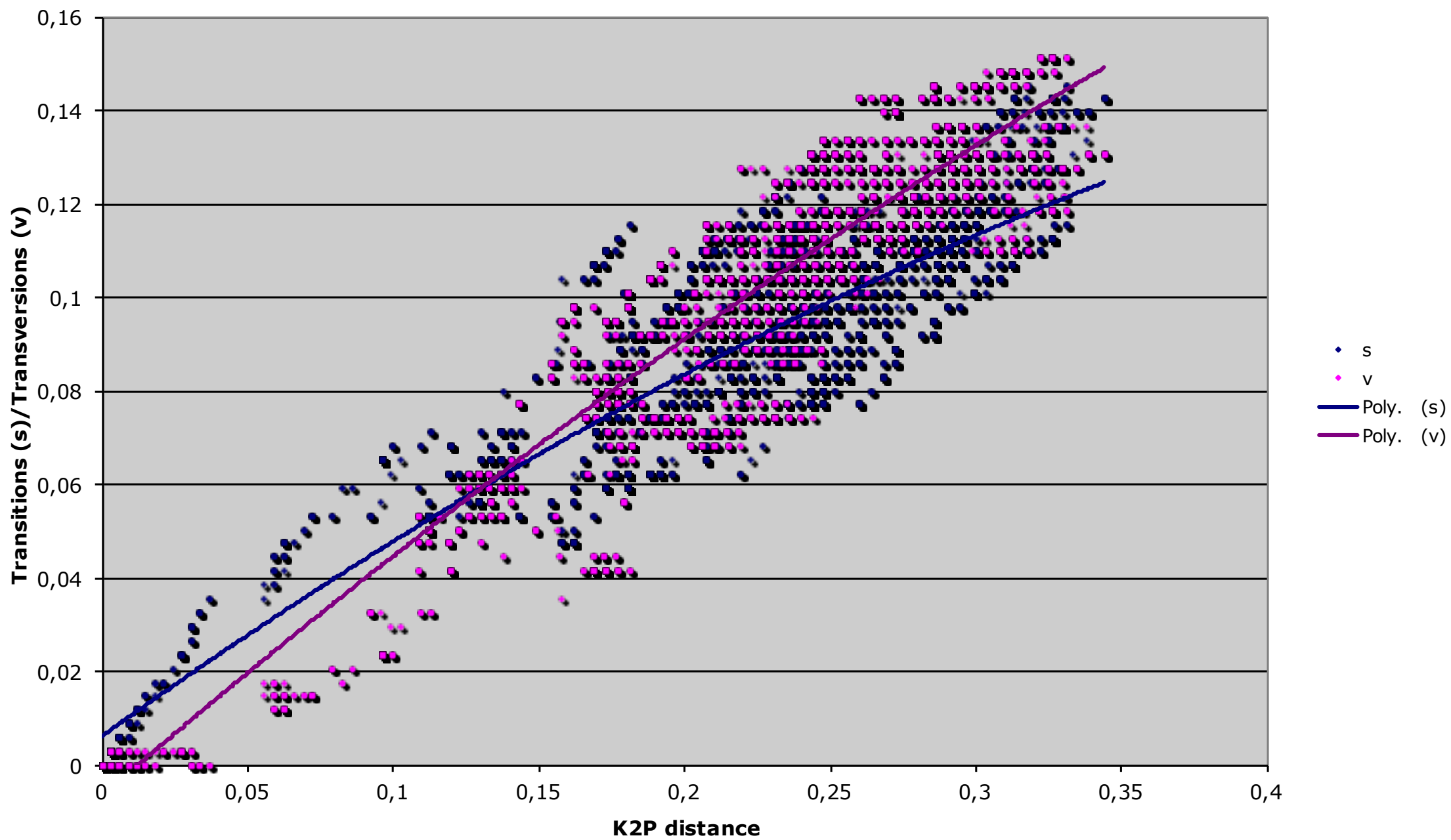

# ND1 1st + 2nd Pos.

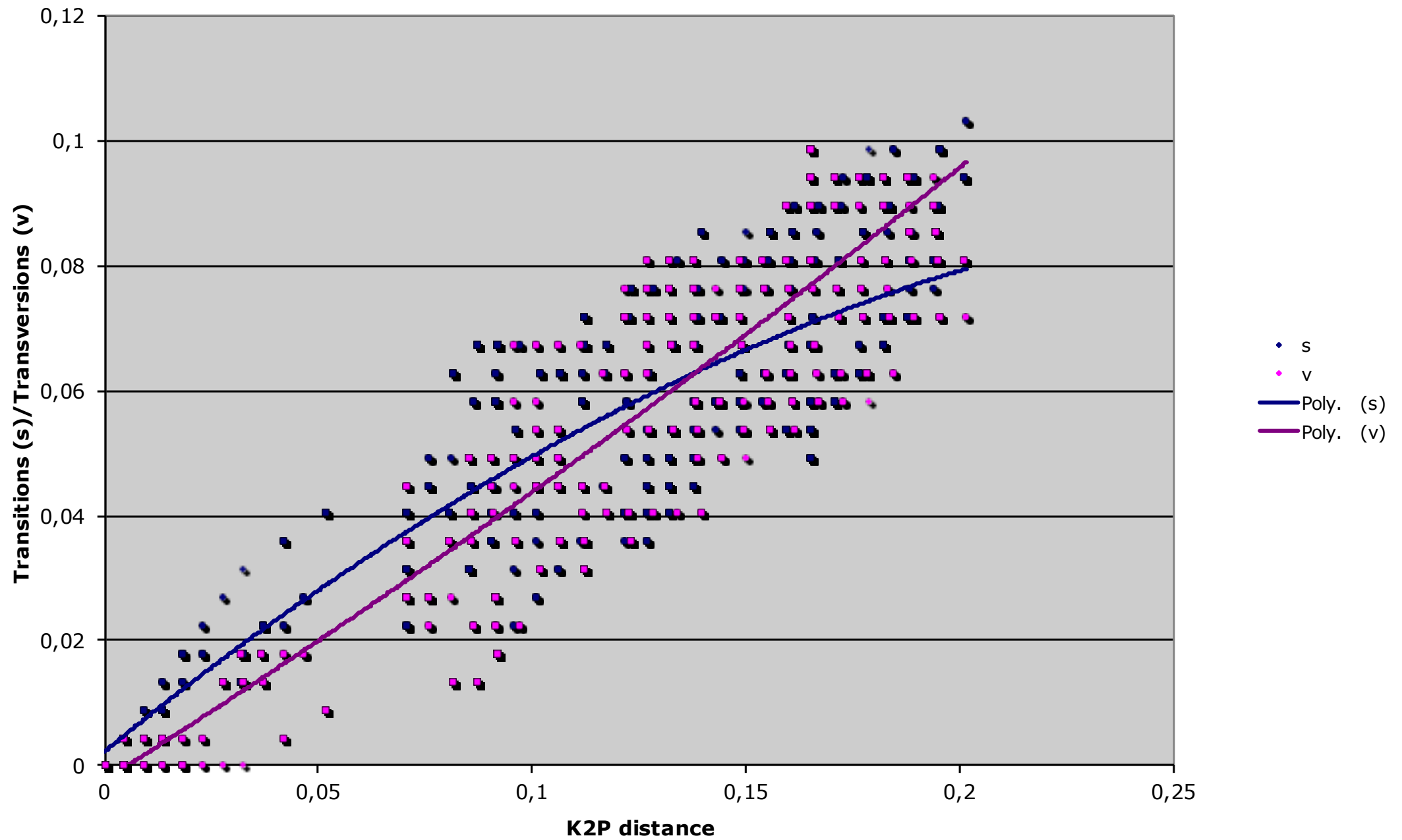

### Folmer 3rd Pos.

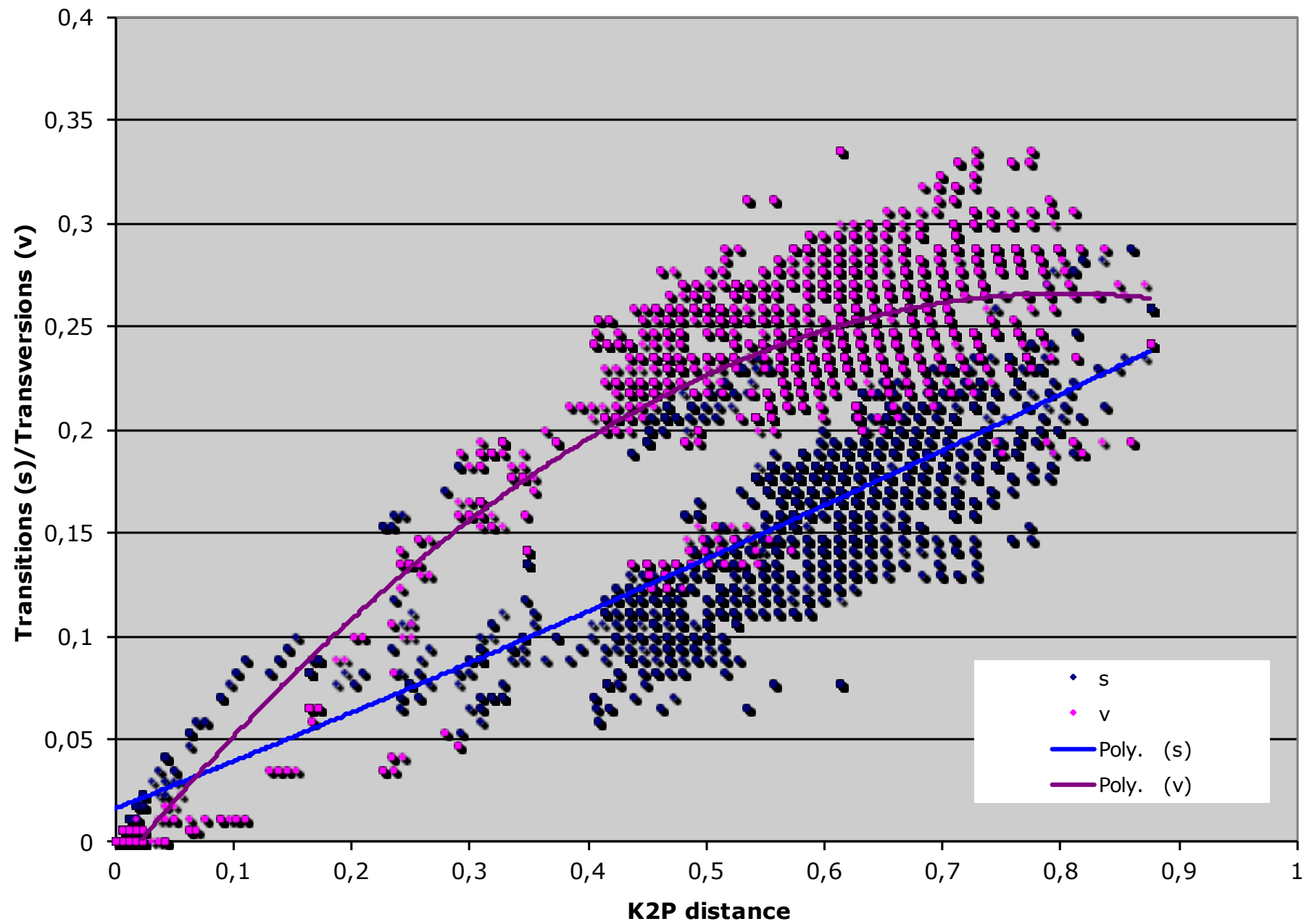

### CO1B 3rd Pos.

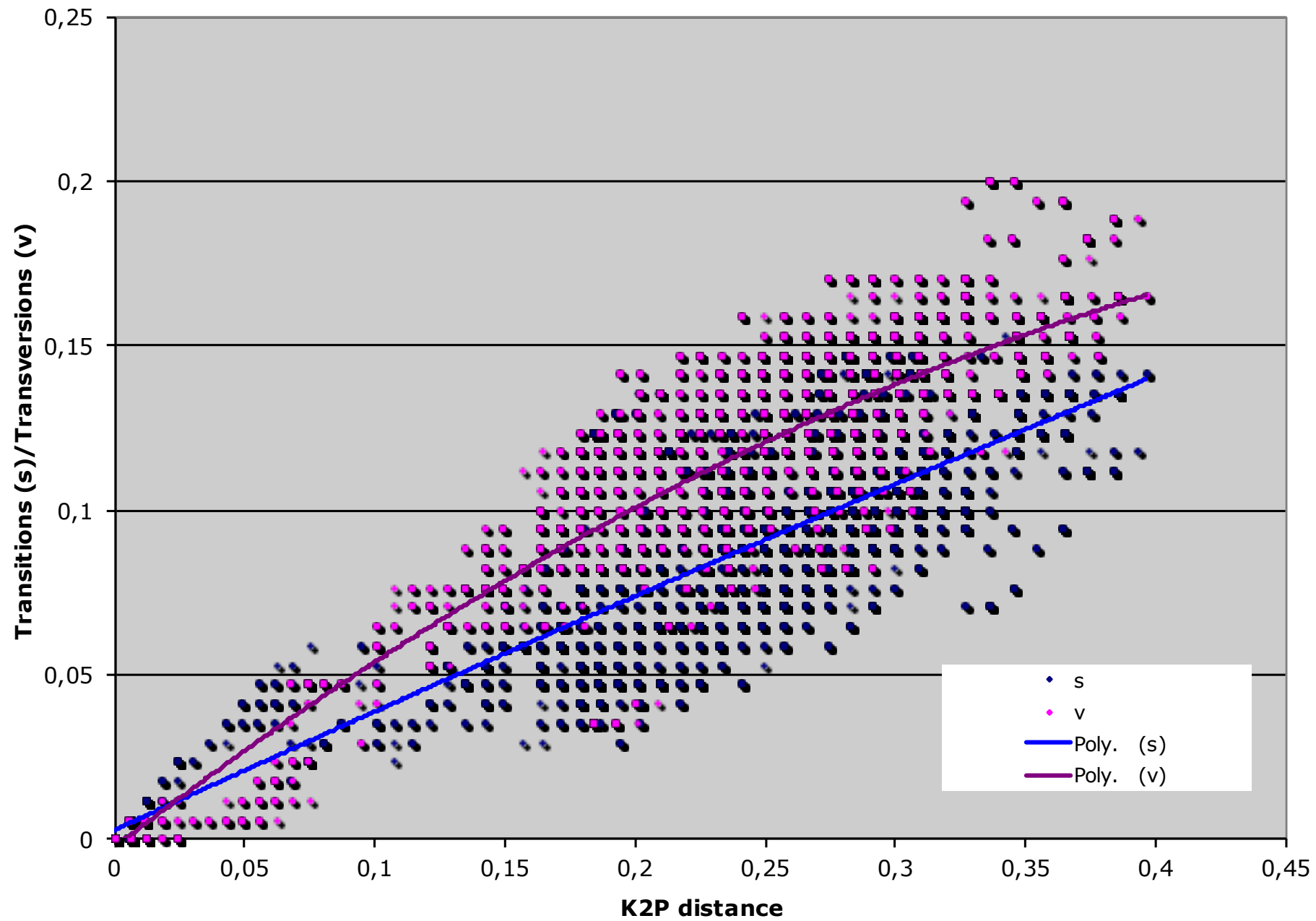

# ND1 3rd Pos.

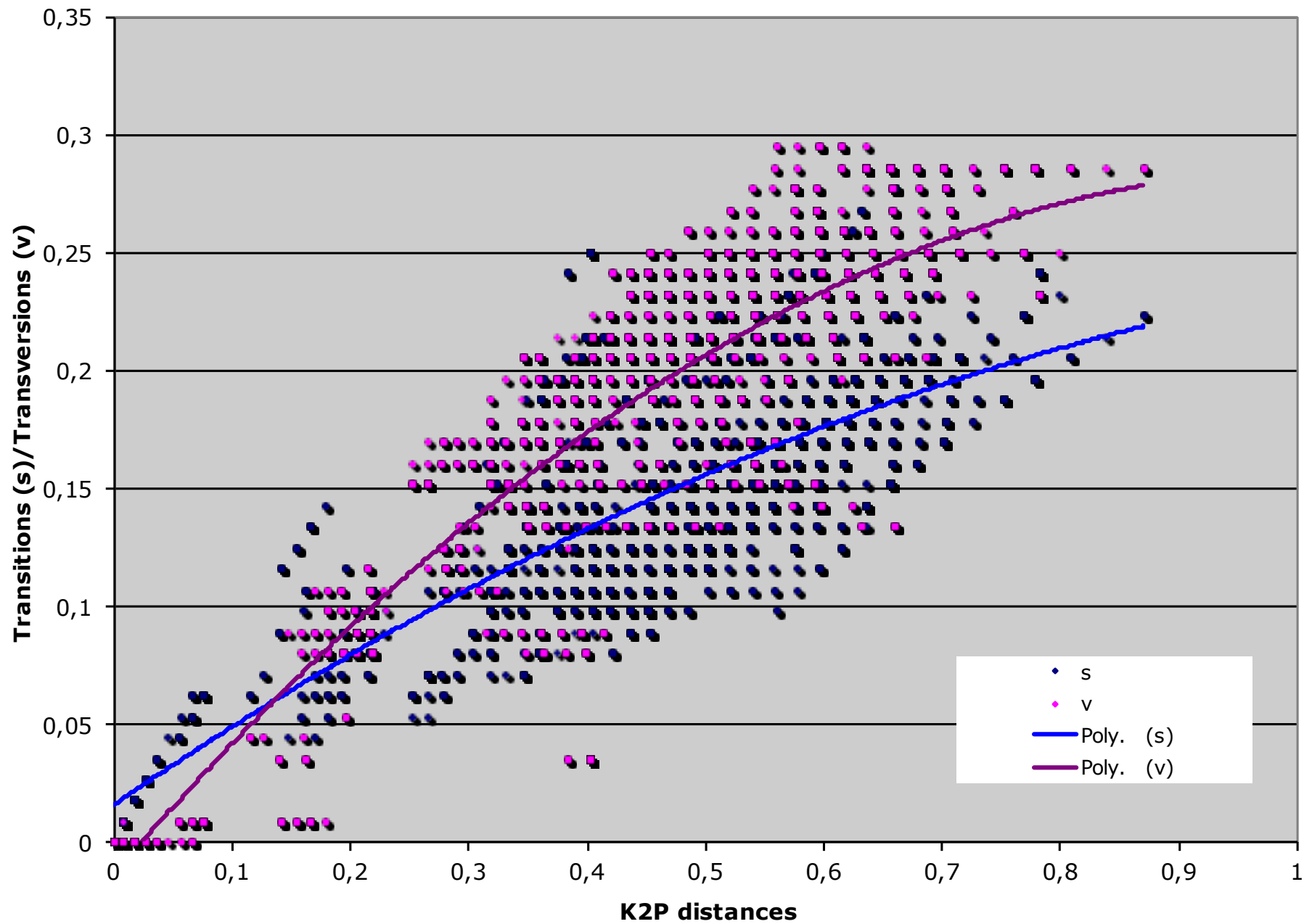

Supplement: S1 Fig — Transition and transversion data of each saturation plot. (PDF) [file pone.0174842.s001.pdf]
